# Unpacking commercial sector opposition to European smoke-free policy: lack of unity, ‘fear of association’ and harm reduction debates

**DOI:** 10.1136/tobaccocontrol-2014-052007

**Published:** 2015-06-08

**Authors:** Heide Weishaar, Amanda Amos, Jeff Collin

**Affiliations:** 1 MRC/CSO Social and Public Health Sciences Unit, University of Glasgow, Glasgow, UK; 2 Centre for Population Health Sciences, University of Edinburgh, Edinburgh, UK; 3 Global Public Health Unit, School of Social and Political Science, University of Edinburgh, Edinburgh, UK

**Keywords:** Tobacco industry, Public policy, Secondhand smoke, Harm Reduction, Advocacy

## Abstract

**Objective:**

Tobacco companies have made extensive efforts to build alliances against comprehensive smoke-free legislation. This article analyses the interaction between actors who opposed the development of the European Council Recommendation on smoke-free environments.

**Methods:**

Drawing on data from 200 policy documents and 32 semistructured interviews and using qualitative textual analysis and organisational network analysis, opponents’ positions on, and responses to, the policy initiative, strategies to oppose the policy, and efforts to build alliances were investigated.

**Results:**

The non-binding nature of the policy, scientific evidence and clear political will to adopt EU-wide measures combined to limit the intensity of commercial sector opposition to the comprehensive EU smoke-free policy. Most tobacco companies, led by the Confederation of European Community Cigarette Manufacturers (CECCM), voiced reservations against the proposal, criticised the policy process and fought flanking measures on product regulation. However, some companies focused on instigating harm reduction debates. These divergent approaches and the reluctance of other commercial actors to demonstrate solidarity with the tobacco sector prevented the establishment of a cohesive commercial sector alliance.

**Conclusions:**

The comparatively limited opposition to EU smoke-free policy contrasts with previous accounts of tobacco industry resistance to tobacco control. While context-specific factors can partially explain these differences, the paper indicates that the sector's diminished credibility and lack of unity hampered political engagement and alliance building. Industry efforts to emphasise the benefits of smokeless tobacco during smoke-free policy debates highlight the potential of harm reduction as a gateway for tobacco companies to re-enter the political arena.

## Background

Since Ireland's comprehensive smoke-free policy in 2004, substantial progress has been made across the EU in protecting citizens from secondhand smoke (SHS) in public places. Corresponding national policy debates have confronted industry lobbyists with an unprecedented challenge,^1^ leading to tobacco companies contesting scientific evidence,^2^ undermining policy development^3^ and mobilising opposition among smokers’ rights groups,^4^ trade unions,^5^ hospitality organisations^6^ and companies focused on technical responses to SHS exposure.^7^

This article analyses the responses of tobacco companies, tobacco-related and other commercial actors^i^ to the development of the Council Recommendation for smoke-free environments, which recommends that member states “provide effective protection from exposure to tobacco smoke in indoor workplaces, indoor public places, public transport and, as appropriate, other public places”.^8^ This non-binding policy was the EU's endorsement of comprehensive national smoke-free policies and the WHO's Framework Convention on Tobacco Control (FCTC) Article 8 guidelines. Initiated in 2007 by a European Commission Directorate General for Health and Consumers’ (DG SANCO) Green Paper,^9^ negotiations took almost 3 years. Following consultations with stakeholders and an impact assessment, the Council Recommendation was adopted in 2009.^8^ This paper investigates opposition to the initiative, interactions between tobacco company representatives, tobacco-related actors and other commercial actors, efforts to build alliances and barriers encountered in seeking to influence the policy process. As the first EU tobacco control initiative adopted following FCTC ratification, the Council Recommendation provides an interesting opportunity to critically examine commercial sector engagement in EU tobacco control policy and efforts to prevent industry interference after international endorsement of FCTC Article 5.3 (which requires all parties to the treaty to protect policies on tobacco control “from commercial and other vested interests of the tobacco industry”^10^).

## Methods

The study used documentary and interview data to investigate opposition to comprehensive EU smoke-free policy. Approximately 200 publicly available documents were reviewed, including policy drafts, minutes of meetings, consultation submissions, briefings and reports. Documents offering indepth information about stakeholders’ views were thematically analysed. To investigate interactions between the various actors opposing EU smoke-free policy, all organisational responses submitted to the Commission's public consultation on smoke-free environments (n=176) underwent a structural network analysis, including those from 35 organisations which the Commission categorised as tobacco-related organisations. Each organisation was registered as a node and assigned attributes, including organisation type and position on the proposal. Two organisations were recorded as having a relationship if: (1) A was mentioned as a collaborating partner on the website or consultation submission of B; and/or (2) A cited three or more references in its submission which were also cited by B; and/or (3) plagiarism detection software showed that A's submission was at least 40% identical to B's. Data were analysed using UCINet V.6^11^ and graphically depicted using NetDraw.^12^ To divide the main component of the network into groups based on their connectedness, the Girvan Newman algorithm was applied.^13^ This identified two distinct components which, based on analyses of node characteristics and interview data, clearly represented two distinct communities. All organisations in the larger component (n=64) were health-related and supported comprehensive EU smoke-free policy, whereas the smaller component (n=24) comprised of tobacco industry organisations that opposed the initiative and argued for extensive exemptions.^14^

Qualitative data were gathered through semistructured, narrative interviews with 35 individuals involved in negotiating the Recommendation. Interviewees were purposively sampled from a list of key individuals identified via the documentary review. Forty eight individuals were contacted, of which six declined and five did not respond. The final sample included decision makers (politicians, civil servants, n=5), representatives of health advocacy organisations (n=13), professional organisations (n=1), scientific institutions (n=4), social partner organisations (trade associations, trade unions, n=4), the tobacco sector (n=4), ventilation industry (n=1) and other commercial sector bodies (n=3). Based on documentary review and informal pilot discussions, an interview topic guide was developed, combining a narrative part and open-ended questions. Interviews were conducted by HW between March and July 2011, asking interviewees to recall experiences of engaging in a process undertaken 5–8 years previously. Twenty two interviewees, who wanted to remain anonymous, suggested descriptors for use in publications, with the desire for anonymity reflected in some vague descriptors used below. Interviews were recorded and transcribed verbatim, except interviews with two tobacco industry representatives who preferred that the interviewer took notes. Using QSR NVivo,^15^ a hermeneutic analytical procedure was followed entailing iteratively identifying themes, coding and thematically analysing data.^16^ Codes were repeatedly compared until analysis of additional interviews did not generate substantively deeper insights. Ethical approval was obtained from the Research Ethics Committee, School of Health in Social Science, University of Edinburgh. A previous publication provides a detailed account of study design and methodological approach.^17^

With high non-response and rejection rates among tobacco industry representatives (4 and 6, respectively, of 14 contacted), and despite intensive recruitment efforts, only four interviewees could provide internal insights into the industry's responses. This may reflect reluctance to engage with a researcher who had previously examined industry efforts to obstruct policy.^18–20^ The analysis, therefore, draws on third parties’ observations of tobacco industry actions. Interviews with other commercial sector representatives, particularly those linked with tobacco-related organisations, provided particularly useful information about opposition to EU smoke-free policy.

## Findings

### Tobacco industry opposition: absence of a unified response

Contrasting fierce industry opposition against national smoke-free policies,^3^ ^4^ ^6^ ^21^ ^22^ tobacco industry representatives reported that there “had not been much commitment” and the industry's response to the initiative had not produced “the well-oiled lobbying campaign that you might think it would have been”. These reports were corroborated by interviews with health advocates who described tobacco industry lobbying as limited, and the comparatively few consultation submissions from tobacco-related organisations (n=35, compared to 81 submissions from health-related organisations).^23^ The analysis, however, provided clear evidence of resistance to comprehensive EU smoke-free policy. Below, we distinguish between (1) tobacco company representatives, (2) tobacco-related actors (other than tobacco companies) with commercial interests in tobacco consumption and (3) non-tobacco or other commercial actors with primary interests beyond tobacco. Table 1 lists all actors identified as opposing comprehensive EU policy, according to their links with tobacco companies.

**Table 1:** Tobacco company representatives, actors with commercial interests in tobacco consumption and non-tobacco actors involved in negotiations

	Tobacco company representatives	Actors with commercial interests in tobacco	Non-tobacco actors
Public collaboration (eg, joint submission) with (other) tobacco companies	Confederation of European Community Cigarette Manufacturers British American Tobacco Japan Tobacco International Imperial Tobacco Group Gallaher		
Apparent collaboration (ie, strong similarity between submissions) with (other) tobacco companies	European Smoking Tobacco Association Gunnar Stenberg AS BAT Malta BAT Cyprus Estonian TMA Irish TMA Latvian TMA Lithuanian TMA Finnish TMA Hungarian TMA Gallaher Norway AS	Imported Tobacco Products Advisory Council UK	
Demonstrable links (eg, information exchange, informal meetings, financial links, membership) with tobacco companies		Freedom Organisation for the Right to Enjoy Smoking Tobacco European Tobacco Wholesalers Tobacco Workers Alliance UK	European Federation of Food, Agriculture and Tourism Trade Unions BusinessEurope German Employers’ Confederation
No demonstrable links with (other) tobacco companies	International Smokeless Tobacco Company Inc Philip Morris International		European Alliance For Technical Non-smoker Protection

BAT, British American Tobacco; TMAs, tobacco manufacturers’ associations.

**Figure 1:**
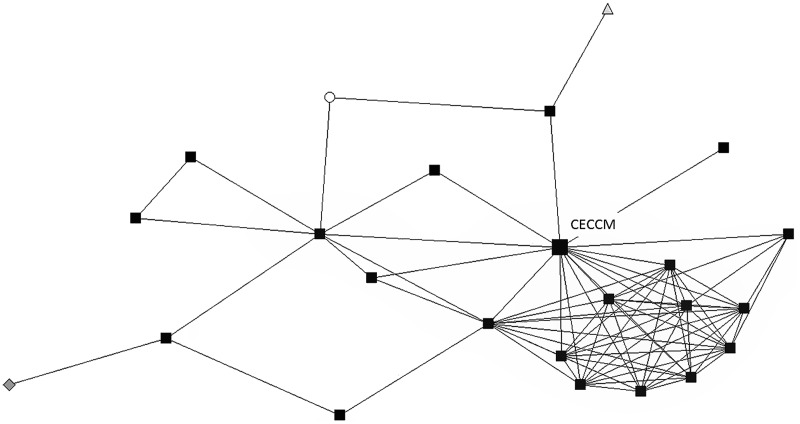
Actors who opposed comprehensive EU smoke-free policy and their relationships with each other; Black square: tobacco manufacturer, White circle: tobacco wholesaler, Light grey triangle: tobacco trade union, Dark grey diamond: social partner organisation.

While various actors publicly voiced reservations about comprehensive smoke-free policy, the data suggest that the strongest counterarguments originated from tobacco manufacturers and tobacco-related actors. They advocated for an “EU-wide smoking ban with exemptions”, designated smoking rooms and exclusion of hospitality venues, private clubs, research laboratories and residential places.^24^ This preference resembled some member states’ policies, which were difficult to enforce and provided loopholes by allowing smoking in multiple public venues.^3^ ^25^ Several tobacco companies, including British American Tobacco (BAT), Japan Tobacco International (JTI), Imperial Tobacco Group (ITG), Gallaher, Ritmeester, Gunnar Stenberg, and European and national tobacco manufacturers’ associations (TMAs), formed the core opposition. The plagiarism detection analysis showed that the wording of submissions from the Confederation of European Community Cigarette Manufacturers (CECCM, a European TMA representing companies including BAT, Gallaher, ITG and JTI), Gallaher Norway AS, Gunnar Stenberg AS, BAT Cyprus and the Estonian, Irish, Latvian and Lithuanian TMAs were almost identical (96–100% similarity according to Turnitin's index of originality). Submissions of the European Smoking Tobacco Association (ESTA), BAT Malta, and Finnish and Hungarian TMAs also showed very strong similarities (66–95%) with each other and the above organisations. An interviewee from a tobacco wholesaler reported that tobacco-related actors had exchanged draft texts and held “short rounds of agreements…and a vote” to achieve a common position and agree on a shared strategy to oppose the EU initiative. Joint actions were described as being led by CECCM in Brussels, which urged national TMAs to submit identical responses to achieve a ‘multiplier effect’ (tobacco wholesaler representative) and demonstrate strong and broad opposition. CECCM's centrality (degree centrality=16), which was considerably higher than that of other alliance members, and the alliance's comparatively high centralisation (44.7%) and compactness (0.58) scores confirmed CECCM's prominent status, positional advantage and ability to manage the hierarchically structured alliance. The sociogramme of opponents who submitted responses to the consultation (figure 1) illustrates the close collaboration between tobacco manufacturers and CECCM's central position within the industry alliance.

**Figure 2:**
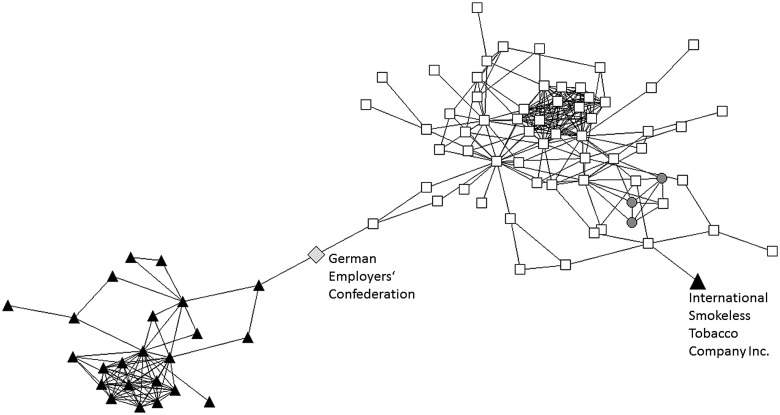
All actors who submitted a response to the European Commission consultation on smoke-free environments and their relationships with each other (main component only); Black triangle: tobacco-related actors, White square: health-related actors, Dark grey circle: local authorities, Light grey diamond: social partner organisation.

Despite efforts to advance a unified response, tobacco companies seemingly struggled to fully align their positions. Contrasting the CECCM-led alliance, Philip Morris’ (PMI) response supported “a total smoking ban…in general public indoor spaces, such as stores, banks, hospitals, public buildings, and public transportation” without suggesting exemptions for specific venues.^26^ Mirroring PMI's attempts to argue for an abolition of the ban on snus under the revision of the EU tobacco products directive in 2010.^27^ PMI used the smoke-free debate as an opportunity to promote deregulation of alternative tobacco products. (EU law^28^ prohibits the marketing of oral tobacco across the EU apart from in Sweden, a ban which has been subject to repeated tobacco industry opposition.^29^) PMI's consultation response advocated product modification as a means to reduce harm, called for consideration of the ‘benefits’ of smokeless tobacco and suggested exemptions to EU smoke-free policy by allowing consumption of non-combustible tobacco products.^26^ Employing similar rhetoric, the International Smokeless Tobacco Company Inc (ISTC), an affiliate of the Altria subsidiary US Smokeless Tobacco Company, highlighted comparatively lower risks of smokeless tobacco and “support in the international public health community for adopting a tobacco harm reduction strategy”.^30^ This depicted agreement with supporters of comprehensive smoke-free policy who saw harm reduction as central to comprehensive tobacco control.^30^ ISTC called on the Commission to introduce “smokeless tobacco availability as a complementary policy option” to EU smoke-free policy.^30^ In the network structure analysis, PMI emerged as an outlier (ie, a member of neither the tobacco industry or tobacco control alliances), whereas the ISTC's position mirrored its unique location as the only tobacco industry actor in an alliance otherwise comprised of organisations with clear interests in health and tobacco control (figure 2).

Complementing documentary data, the interviews provided evidence of tobacco industry attempts to instigate, join and frame debates on, and direct attention towards, harm reduction. Such debates were conceived as offering unique public relation opportunities to reposition tobacco companies as reasonable and legitimate stakeholders who could add value to the policy process, lobby for removal of restrictions on smokeless tobacco products, and depict harm reduction policies as necessary adjuncts to prohibiting smoking in public places.

Everything the tobacco industry has been doing in the last few years is…trying to find as many policy hooks as they can, including [the Recommendation], in order to build up support for snus as a safer alternative or even as a kind of a smoking cessation aid. (Lobbyist)

While keen to avoid being perceived as strongly opposing a policy aimed at protecting citizens from SHS harm, the CECCM-led tobacco industry alliance worked to hamper the policy process and derail the initiative. CECCM's submission strongly criticised DG SANCO staff for an approach to stakeholder consultations depicted as biased and undemocratic, and instead called for “dialogue and consultation with all interested stakeholders, including the tobacco sector” and for what it framed as a more inclusive approach to policy development.^24^ Representatives of tobacco wholesalers, the cigar industry and smokers’ rights’ organisations followed CECCM's lead in questioning the policy assessment that had been undertaken, arguing that the impact of smoke-free policies could not be accurately and comprehensively assessed.^31–33^ One letter from Roberto Zanni, CECCM's chairman, made available by an interviewee, indicates that efforts to disrupt the policy process culminated in July 2008 when tobacco companies turned to other DGs to denounce DG SANCO's approach. Zanni R. [Letter from Roberto Zanni to Alexander Italianer regarding impact assessment on the follow-up initiative on smoke-free environments]. Personal communication, 2008. The letter to Alexander Italianer, Deputy Secretary General of the European Commission and Chairman of the European Commission Impact Assessment (IA) Board, argued that “some of the methodological approaches employed in conducting the IA may not be entirely in line with the EU's IA Guidelines” and questioned whether DG SANCO's IA complied with the European Commission's Better Regulation strategy.

Finally, interviews revealed considerable increases in tobacco companies’ activity in the closing stages of the process. This intensification was triggered by the unexpected inclusion in the policy draft of calls to revise the Tobacco Products Directive and assess future EU policies regarding plain packaging and graphic health-warning labels.^34^ One health advocate reported that these flanking measures had been strategically inserted by representatives of governments supporting strong EU policy, calculating that late insertion would deprive opponents of time to devise an effective strategic response. According to a tobacco wholesaler representative, the industry was surprised by this move and unable to mobilise sufficient opposition to the proposed flanking measures. Despite “a huge amount of industry lobbying trying to keep all mention of plain packaging out of this recommendation” (public health advocate), industry representatives were unable to prevent passage of the amended text.

### Factors reducing opposition to comprehensive EU smoke-free policy

In fighting comprehensive smoke-free policy, opponents argued that health risks from SHS were ‘relatively minor’,^35^ insufficiently proven^33^ ^36^ ^37^ or unfounded.^32^ ^38^ Interviews with public health advocates, however, emphasised ‘conclusive evidence’ and ‘strong scientific consensus’ about the detrimental impacts of SHS and health benefits of comprehensive smoke-free policies as significantly impeding opposition. Policy evaluations in EU member states were employed by health advocates to highlight the feasibility, effectiveness and acceptability of comprehensive smoke-free policies and the inadequacy of partial measures.^39–42^ The strong evidence base and momentum in adopting national smoke-free legislation seemed to mobilise decision makers to adopt measures at EU level. Exposure of previous tobacco industry strategies was used to warn decision makers against industry interference, with advocates reporting that industry representatives were “trotting out the same arguments that they had trotted out in all the other countries where smoke-free legislation had been enacted”, so supporters “didn't really have a very difficult job to do in rebutting the industry”.

These factors, however, only partially explain the limited efforts of opponents in countering the development of the Recommendation. Arguably as important was the EU's restricted scope to adopt binding smoke-free legislation due to its lack of legal remit to harmonise national public health legislation, such that opponents did not perceive the initiative as a substantial threat. One tobacco industry representative, for example, reported that “the pressure came off the cooker” and considerable downscaling of lobbying efforts as soon as it was clear that binding legislation would not be supported in the Council of the EU. Similarly, representatives of other industries reported that the Recommendation was perceived as non-threatening and of a low priority, reflecting the implausibility of legal consequences and low probability of translation into national legislation.

### Barriers to building an alliance against comprehensive EU smoke-free policy

Notwithstanding their general reticence about increasing EU regulation, membership links and previous collaboration with tobacco companies, the data indicate limited opposition to comprehensive EU policy from social partners and pan-European trade associations, like BusinessEurope (an organisation representing industry associations across Europe), the European Trade Union Confederation (ETUC), the European Federation of Food, Agriculture and Tourism Trade Unions (EFFAT), and the European trade association representing hotels, restaurants and cafés (HOTREC).^43^ ^44^ In contrast to previous tobacco industry success in building opposing alliances with like-minded organisations,^19^ ^45^ only one connection was identified between a tobacco-related actor and a non-tobacco actor (the German Employers’ Confederation, figure 1). Documentary data were supported by interview accounts, revealing that interactions between tobacco companies and representatives of other sectors were largely confined to information exchange. Reflecting on the “fear of association” and the reluctance of other commercial actors to publicly collaborate with tobacco companies, tobacco industry representatives confirmed that their attempts to build alliances were frequently rejected, resulting in “restricted room for manoeuvre” (tobacco wholesaler representative) and limited opportunities to counter the policy.

In addition to the low priority afforded to the initiative by pan-European organisations given the unlikelihood of binding legislation, structural features of EU policymaking seemed to further hamper alliance building. Pan-European commercial sector organisations struggled to reach consensual positions on the policy proposal, with several interviewees reporting cumbersome discussions. A tobacco industry representative, for example, reported that HOTREC had been “between a rock and a hard place” in balancing views of national members opposing the policy (primarily organisations from states with no or partial national legislation) and those willing to support the EU initiative (usually organisations from states with comprehensive legislation). Reflecting on similar difficulties of aligning diverse members, a representative of another umbrella organisation reported that having “to coordinate things with everyone…[often led to]…very general and…non-committal” positions.

Pan-European organisations also seemed to carefully weigh the benefits of opposing a specific EU-level initiative against a more general, long-term aspiration to be perceived as reasonable, progressive and constructive stakeholders, supportive of EU-wide cooperation. Strategic considerations about the impact of their responses on their public image and desires to not appear as “a kind of old-school, old type” but “modern” business (social partner representative) influenced organisational decisions on whether to demonstrate agreement with tobacco-industry organisations. While reporting that they had generally been opposed to comprehensive EU smoke-free policy and met with tobacco company representatives to exchange information and coordinate responses, several social partners and non-tobacco trade representatives recounted a reluctance to position themselves as tobacco industry allies. It is important to note that the guidelines to FCTC Article 5.3 were adopted at the third session of the Conference of the Parties in November 2008, that is, when EU smoke-free policy was negotiated.^46^ Aware of such discussions, other commercial sectors seemed keen to distance themselves from the tobacco industry and portray themselves as legitimate stakeholders, who took “balanced” (social partner representative) positions on EU policies. A representative of one pan-European umbrella business organisation with tobacco company membership, for example, was eager to highlight that tobacco industry members did not “punch above their weight” and that the organisation was “not there only to represent the tobacco industry”. Contrasting the tobacco industry's use of companies which focused on technical solutions to reducing SHS exposure as surrogates in national smoke-free policy debates,^7^ ^47^ a representative of the European Alliance for Technical Non-Smoker Protection (EATNP) reported that some EATNP members had refused any contact with tobacco industry representatives because “they want to avoid that they are, from an image point of view, shuffled onto the side of the cigarette industry”. Instead, ventilation companies had sought to portray themselves as intermediaries in the policy debates which were “situated right in the middle” between the tobacco industry and public health organisations, providing protection from SHS while allowing smoking in public places (EATNP representative). Owing to the wide-spread perception of ventilation companies as tobacco industry allies, EATNP had, however, faced considerable constraints when attempting to collaborate with public health representatives.

## Discussion

Previous research extensively documents tobacco industry efforts to combat tobacco control policies, including national smoke-free policies and the regulation of tobacco advertising and tobacco products at EU level.^3^ ^48^ WHO Director General Margaret Chan has expressed anticipation of “well-orchestrated, well-funded, and aggressive resistance every step along the way” as further tobacco control policies are adopted.^49^ By analysing commercial sector opposition to EU smoke-free policy and showing that tobacco company representatives and other commercial actors carefully focused their lobbying on specific aspects of the policy draft and process, this paper shows that the legislative context strongly influences the dynamics and intensity of industry opposition.

Two context-specific factors are particularly important in interpreting the findings. First, the interview data show that the non-binding nature of the proposed policy considerably shaped opponents’ responses to the proposal. The fact that the EU is not granted competence to harmonise national public health legislation^50^ and its inability to adopt binding, enforceable legislation aimed at protecting citizens from SHS seemed to considerably reduce interest in, and opposition to, the initiative. Given that non-binding EU policy was not perceived as a significant threat to their business, tobacco industry and (arguably more important in terms of building broader alliances) other commercial sector actors did not strongly oppose its adoption. The policy's focus on protecting vulnerable groups from the detrimental effects of SHS exposure,^9^ combined with substantial evidence on the health impacts and the effectiveness of comprehensive smoke-free policies, appeared to further reduce opponents’ motivation and opportunities to counter EU level action. These findings contrast literature outlining the industry's fierce opposition to national smoke-free policies and recent EU tobacco product regulation and trade-related tobacco control measures.^3^ ^51^ Contrasting the small number of consultation submissions countering the European Commission's proposal to tackle SHS exposure, the recent consultation on the EU Tobacco Products Directive generated over 2300 industry submissions, most voicing strong reservations about the legally binding policy document.^51^ Multiple articles, which report fierce opposition to national smoke-free legislation, provide further evidence of continuing tobacco industry opposition to binding, enforceable tobacco control policy.^2^ ^3^ ^6^ ^21^ ^22^ ^52–56^ By emphasising the importance of the legislative context and policy specific factors, this paper contributes to the existing literature which reflects on the industry's strategic adaptation to different political realities,^57^ ^58^ highlighting the need for nuanced and contextual analyses of tobacco industry opposition. It also reminds those with an interest in effective tobacco control that opponents will respond most forcefully to policies that are most likely to be effective and pose the highest threat to commercial interests.

While the importance of context-specific factors means that the findings cannot be generalised, our study provides valuable insights into the complexities of tobacco companies’ engagement with each other and other sectors, the dynamics which shape opposition to tobacco control policy at EU level, and the impediments to developing effective EU tobacco control policy. Our indepth analysis of the diverse approaches which opponents pursued in countering comprehensive EU smoke-free policy highlights the contrast between some tobacco companies’ decision to overtly oppose comprehensive smoke-free policy and the seemingly more circumscribed opposition by other actors. The findings demonstrate the difficulties opponents faced when justifying their resistance to EU smoke-free policy and their limited success in reiterating well-known arguments and tactics to counter tobacco control. The CECCM-led alliance subsequently focused its efforts on disputing any reference in the policy document to binding EU level tobacco regulation, arguing for more consultations with tobacco industry representatives, criticising DG SANCO's approach to policy development and instigating division among European Commission DGs. Importantly, the findings point to a considerable reluctance among non-tobacco actors, notably pan-European organisations representing the commercial sector, to openly join such opposition and publicly counter the policy proposal. This contrasts with historical accounts depicting umbrella organisations as serving tobacco industry interests^19^ and commercial actors as willing to ally with tobacco companies in opposing regulation,^59^ possibly indicating increasing commercial actors’ concern about tarnishing their public image through agreeing with tobacco industry interests and reluctance to ally with tobacco companies. Our analysis also shows that tobacco companies contested DG SANCO's approach to guarding against tobacco industry interference in the policy development and that tobacco control advocates’ rhetoric on the need to protect tobacco control from tobacco industry interests was crucial in isolating the industry. This analysis testifies to the significance of FCTC Article 5.3 and its considerable potential to hamper industry influence on policymaking and strengthen tobacco control.

Complementing previous research, which analyses tobacco companies’ differential political positions and strategies in opposing tobacco taxation in the Czech Republic,^58^ this study highlights the different approaches which PMI, ISTC and the CECCM-led alliance pursued in countering the policy proposal. Consistent with recent industry efforts to use harm reduction to increase corporate credibility and improve access to policymakers,^60^ PMI and ISTC focused on directing political attention to the potential role of smokeless tobacco in alleviating SHS harms. Our study covers a time period (2006–2009) when tobacco companies were investing extensively in smokeless tobacco and starting to publicly engage in harm reduction debates.^60^ ^61^ Introducing a harm reduction narrative into the debates on EU smoke-free policy seemed to be a strategic move aimed at creating opportunities to present themselves as socially responsible corporations and legitimate political stakeholders. Our analysis, therefore, provides early evidence of tobacco companies’ strategic thinking and suggests that tobacco companies seized debates on EU smoke-free policies to highlight purported benefits of, and develop their narrative about, harm reduced tobacco products and promote policies which would facilitate market access of these products. Recent industry responses to the revised EU tobacco products directive show continuity in claims that alternative tobacco products are effective aids when reducing or quitting smoking and that the EU inhibits development of less harmful products by banning certain forms of tobacco.^51^ The presented evidence of PMI's and ISTC's strategic use of harm reduction discourse to publicly depict common ground with tobacco control goals is reiterated by research indicating that harm reduction debates assist tobacco companies in demonstrating alignment with public health interests and rebuilding of their corporate legitimacy.^60^ It thus corroborates concerns that such debates might help tobacco companies to overcome their relative isolation and re-enter the policy arena, undermining FCTC Article 5.3.^60^

Our study is, to the best of our knowledge, one of three^60^ ^58^ which present data from interviews with tobacco industry representatives to investigate industry opposition to tobacco control. While we acknowledge both the limitations which inevitably arise from the small number of industry respondents and the issues associated with engaging with tobacco industry representatives, the interviews with tobacco industry representatives and other opponents generated valuable information which would not have otherwise been obtained. This study shows that explicitly seeking the views of those opposed to tobacco control, rather than relying on documentary data or third party accounts, can help to develop a nuanced and comprehensive understanding of opposition, intraindustry dynamics and the complexities of tobacco companies’ political engagement and alliance building.

## Conclusion

Our findings highlight the potential oversimplification of portraying diverse tobacco companies and related organisations as a monolithic body. The presentation of such actors as ‘big tobacco’^62^ has served to raise awareness about shared interests in tobacco production and consumption and the undermining of tobacco control, while reference to “the industry and its allies” has highlighted tobacco companies’ successes in working through surrogates and front groups.^58^ Research on the lack of unity between tobacco companies, their varying strategies to engage in policymaking, and the barriers to alliance building with non-tobacco actors illuminates complex dynamics of industry interactions, with potentially significant implications for policy analysis and health advocacy.

**What this paper adds**

- Opposition to EU smoke-free policy was of limited scope and intensity, reflecting the low priority that opponents afforded to the non-binding policy proposal, reluctance to oppose a policy initiative which was clearly framed around public health and strategic efforts to present themselves as constructive stakeholders.
- Tobacco companies pursued different lobbying strategies, with a Confederation of European Community Cigarette Manufacturers (CECCM)-led alliance opposing the policy, and Philip Morris’ (PMI) and International Smokeless Tobacco Company Inc (ISTC) using the debates on EU smoke-free policy to initiate harm reduction debates.
- Hoping to avoid being associated with the tobacco industry and facing difficulties in building consensus among members, pan-European business organisations that had previously allied with the tobacco industry did not publicly oppose EU smoke-free policy.
- By analysing data from interviews with tobacco industry representatives and tobacco control opponents, this paper provides unique insights into the complex dynamics of tobacco control opposition.
- The joint depiction of various tobacco-related actors as ‘big tobacco’ risks over-simplifying the complexities of interaction and alliance building among tobacco control opponents.

## Footnotes

i This follows the European Commission's categorisation of tobacco-related actors (eg, tobacco growers, manufacturers, wholesalers, retailers, trade unions) as having a commercial interest in tobacco consumption, distinguishing them from other commercial, non-tobacco actors and representatives of other industry sectors (eg, hospitality industry representatives, ventilation industry representatives and representatives of general trade associations).
